# Loss of hexokinase 1 sensitizes ovarian cancer to high-dose metformin

**DOI:** 10.1186/s40170-021-00277-2

**Published:** 2021-12-11

**Authors:** Daniela Šimčíková, Dominik Gardáš, Kateřina Hložková, Martin Hruda, Petr Žáček, Lukáš Rob, Petr Heneberg

**Affiliations:** 1 Third Faculty of Medicine, Charles University, Ruská 87, CZ-100 00 Prague, Czech Republic; 2 CLIP – Childhood Leukaemia Investigation Prague, Prague, Czech Republic; 3 Department of Pediatric Hematology and Oncology, Second Faculty of Medicine, Charles University, Prague, Czech Republic; 4 University Hospital Kralovské Vinohrady, Prague, Czech Republic; 5 Faculty of Science, BIOCEV, Charles University, Vestec, Czech Republic

**Keywords:** Aerobic glycolysis, Hexokinase, Metabolism reprogramming, Metformin, Nicotinamide adenine dinucleotide phosphate, Oxidative phosphorylation

## Abstract

**Background:**

Hexokinases (HKs) are well-studied enzymes catalyzing the first step of glycolysis. However, non-canonical regulatory roles of HKs are still incompletely understood. Here, we hypothesized that HKs comprise one of the missing links between high-dose metformin and the inhibition of the respiratory chain in cancer.

**Methods:**

We tested the isoenzyme-specific regulatory roles of HKs in ovarian cancer cells by examining the effects of the deletions of HK1 and HK2 in TOV-112D ovarian adenocarcinoma cells. We reverted these effects by re-introducing wild-type HK1 and HK2, and we compared the HK1 revertant with the knock-in of catalytically dead HK1 p.D656A. We subjected these cells to a battery of metabolic and proliferation assays and targeted GC×GC-MS metabolomics.

**Results:**

We found that the HK1 depletion (but not the HK2 depletion) sensitized ovarian cancer cells to high-dose metformin during glucose starvation. We confirmed that this newly uncovered role of HK1 is glycolysis-independent by the introduction of the catalytically dead HK1. The expression of catalytically dead HK1 stimulated similar changes in levels of TCA intermediates, aspartate and cysteine, and in glutamate as were induced by the HK2 deletion. In contrast, HK1 deletion increased the levels of branched amino acids; this effect was completely eliminated by the expression of catalytically dead HK1. Furthermore, HK1 revertants but not HK2 revertants caused a strong increase of NADPH/NADP ratios independently on the presence of glucose or metformin. The HK1 deletion (but not HK2 deletion) suppressed the growth of xenotransplanted ovarian cancer cells and nearly abolished the tumor growth when the mice were fed the glucose-free diet.

**Conclusions:**

We provided the evidence that HK1 is involved in the so far unknown glycolysis-independent HK1–metformin axis and influences metabolism even in glucose-free conditions.

**Supplementary Information:**

The online version contains supplementary material available at 10.1186/s40170-021-00277-2.

## Introduction

Ovarian cancer has the highest mortality among gynecological diseases, with almost 300,000 new diagnoses and 185,000 deaths worldwide in 2018 [1]. Due to non-specific symptoms of ovarian cancer, approximately 80% of women are diagnosed at advanced stages of the disease, when the cancer cells had already spread beyond the ovaries through the peritoneum, particularly to the omentum [2]. The usual first-line treatment of patients with advanced ovarian cancer consists of surgical reduction of the tumor mass, followed by multiple cycles of paclitaxel and carboplatin. Alternatively, three cycles of chemotherapy may be conducted prior to the surgery [3]. The survival of patients with BRCA1/2-positive ovarian tumors is improved when treated with PARP inhibitors [4]. Recently, two metabolically different groups were identified within the same histopathological type of ovarian cancer (high-grade serous ovarian carcinoma, HGSOC), which emphasized the importance of reflecting and targeting cancer metabolism. Importantly, HGSOC with the preference for oxidative phosphorylation exhibited an increased response to conventional chemotherapies, unlike HGSOC with the preference for aerobic glycolysis [5].

Reprogrammed metabolism represents one of the key cancer hallmarks [6]. Many tumors rely on aerobic glycolysis as noticed already by Otto H. Warburg almost 100 years ago [7]. In the present study, we focus on hexokinases (HKs), which catalyze the first irreversible step of glycolysis. Mammalian cells express four isoenzymes of ATP-dependent hexokinases, HK1-4, although their presence and expression levels differ among tissues [8]. HK1-3 are structurally similar hexokinases with high affinity to glucose (*K*_M_ about 0.02 mM); only the HK4 isoenzyme has a lower affinity to glucose (*K*_M_ about 5 mM), which is related to its glucose-sensing function. The three high-affinity enzymes differ in their subcellular localization. HK1 and HK2 are mostly localized on the outer mitochondrial membrane, HK3 is in a perinuclear compartment [8]. HK2 can translocate between mitochondria and the cytosol depending on glucose, glucose 6-phosphate, and PKB/Akt, regardless of ATP, whereas HK1 remains bound to mitochondria [9]. All the HKs have been found amplified in primary cancer cells. HK2 amplification appears to be related to p53 variations, whereas HK1 and HK3 amplifications are related to amplifications of the oncogenes c-Myc and mouse double minute 2 homolog (MDM2), and deletion of the tumor suppressor cyclin-dependent kinase inhibitor 2A (CDKN2A) [10]. The functions of HKs extend beyond glucose phosphorylation, and the HKs serve as regulatory proteins. HK2 is well-known for its regulatory function in tumorigenesis, thereby being suggested as a preferred isoenzyme for tumors [11–14]. However, the recent report of HK1 as an effector of KRAS4A highlighted the importance of the latter isoenzyme in tumorigenesis [15].

The incidence and treatment of different cancers, including ovarian cancer, have been repeatedly associated with improved outcomes in patients treated with metformin [16, 17]. Metformin, the most widely prescribed drug for the management of type II diabetes, inhibits hepatic gluconeogenesis through non-competitive inhibition of mitochondrial glycerophosphate dehydrogenase [18]. In addition, metformin increases [AMP]/[ADP] and/or [ADP]/[ATP] concentration ratios, leading to the activation of AMP-activated protein kinase (AMPK), or liver kinase B1 (LKB1), leading to a decrease in transcription of genes involved in gluconeogenesis [19–21]. However, the complete mechanism of the metformin action, whether metformin might protect against cancer or treat malignancies, remains unclear. Higher than therapeutic concentrations of metformin inhibit mitochondrial respiratory chain directly; previous studies typically used up to 5 mM metformin in vitro [5, 22, 23].

Since metformin influences energy/mitochondrial metabolism and induces a compensatory increase in glycolysis [24, 25], we hypothesized that the HKs could comprise one of the missing links between the metformin treatment and subsequent energetic stress and metabolism rewiring. On the model of ovarian cancer, we aimed to find out, whether the depletion of HKs that are expressed in ovarian cancer cells has any effect on the effects of metabolic stress, including the respiratory chain inhibition by high-dose metformin treatment. We also tested, whether the metformin action is HK isoenzyme-specific, and focused on mechanisms that stay behind the newly uncovered HK1-metformin axis.

## Materials and methods

### Patients

The resected anonymized high-grade serous ovarian carcinoma tissues and matched normal adjacent ovarian tissues were taken from Czech patients who underwent surgery for clinical purposes in the University Hospital Královské Vinohrady. The dispase-based isolation protocol by Shepherd et al. [26] was followed. The cells were allowed to proliferate in culture for further 2 to 6 weeks. The purity of cultured cells was tested by staining with vimentin-Alexa488 (Cell Signaling Technology, Danvers, MA, #9854), pan-keratin-PE (Cell Signaling Technology, #5075), and smooth muscle actin-Alexa488 (eBioscience, San Diego, CA, #53-9760-82). Due to the age of the examined ovarian cancer patients, the cells from matched normal adjacent tissues consisted of fibroblasts instead of the already mostly atrophied epithels. The use of primary cells was approved by the Ethics Committee of the Third Faculty of Medicine, Charles University (approval not numbered, dated April 29, 2020), and by the Ethics Committee of the University Hospital Královské Vinohrady (approval #EK-VP/24/0/2020, dated June 3, 2020).

### Cell lines

Human ovarian cancer cell lines TOV-21G, TOV-112D, and ES-2 from the ATCC were cultured in the respective ATCC-recommended medium. HEK-293T cells were cultured in high-glucose DMEM supplemented with 10% FBS. Cells were maintained at 37 °C in 5% CO_2_/95% air. Mycoplasma contamination was routinely examined using a qPCR-based service provided by Eurofins Genomics (Ebersberg, Germany). To conduct experiments, DMEM (Thermo Fisher Scientific; Gibco #A14430-01) supplemented with or without 5.5 mM glucose and/or 2 mM glutamine, and 10% FBS was used unless stated otherwise.

### Preparation of knockout cell lines

The knock-outs of HK1 (HK1^−^) and knock-outs of HK2 (HK2^−^) were prepared in the TOV-112D cell line using pSpCas9(BB)-2A-GFP (PX458), which was a gift from Feng Zhang (Addgene plasmid #48138; http://n2t.net/addgene:48138; RRID: Addgene_48138) [27]. The respective oligonucleotides encoding sgRNAs were designed using the CHOPCHOP v3 tool [28] (see Table S1 for the list of primers and sgRNAs) and cloned according to the recommended SAM target sgRNA cloning protocol [29]. The cells were transfected using Lipofectamine 2000 (Invitrogen, Carlsbad, CA) and subjected to a single-cell sorting of the GFP-positive cells by the BD Influx cell sorter (BD Biosciences, San Jose, CA). DNA was isolated from each cell clone using QuickExtract DNA extraction solution (Lucigen, Middleton, WI). The target HK1 and HK2 exons were amplified Herculase II DNA polymerase (Agilent Technologies, Santa Clara, CA). The PCR products were analyzed using a RFLP approach targeting the sgRNA-complementary sequences. The clones showing changes in RFLP compared to control cells were further tested by Western blotting.

### Preparation of knockdown cell lines

The knockdown cell lines were prepared using CRISPR interference with a lentiviral vector pLV hU6-sgRNA hUbC-dCas9-KRAB-T2a-Puro, which was a gift from Charles Gersbach (Addgene plasmid # 71236; http://n2t.net/addgene:71236; RRID:Addgene_71236) [30] and encodes KRAB-dCas9 and the puromycin resistance gene. For cloning of sgRNA-encoding oligonucleotides (see Table S1 for the list of primers and sgRNAs), a lentiviral vector pLKO5.sgRNA.EFS.GFP was used (a gift from Benjamin Ebert (Addgene plasmid # 57822; http://n2t.net/addgene:57822; RRID:Addgene_57822) [31]). The ovarian cancer cell lines were transduced by lentivirus encoding KRAB-dCas9 and puromycin resistance and then selected with puromycin. The respective pools of the selected cells were transduced by lentiviruses containing sequences for sgRNAs and GFP. These pools of GFP-positive cells were sorted and used for subsequent experiments.

### Preparation of HK revertants and HK1 variants

To amplify *HK1* and *HK2* cDNA, the plasmids encoding HK1 and HK2 (both from Origene, Rockville, MD) were used as templates for PCR (Table S1). The amplified PCR products were cloned into pHIV-EGFP, which was a gift from Bryan Welm & Zena Werb (Addgene plasmid #21373; http://n2t.net/addgene:21373; RRID: Addgene_21373) using In-Fusion HD Cloning Plus kit (Takara, Kusatsu, Japan). The HK1^−^ and HK2^−^ TOV-112D cell lines were transduced by lentiviruses encoding HK1 and HK2 and afterwards sorted according to GFP expression. The pools of GFP-positive cells were examined for HK1 or HK2 expression by Western blotting and used for subsequent experiments.

The amplified *HK1* cDNA was cloned into the plasmid pET-28a(+) and variations p.D656A and p.T657A were introduced via site-directed mutagenesis using *Pfu*Ultra High-Fidelity DNA polymerase (Agilent, Santa Clara, CA) (Table S1). The p.D656A variant was further introduced via site-directed mutagenesis into the pHIV-EGFP-HK1 plasmid. To generate the HK1 D656A knock-in, the HK1^−^ TOV-112D cell line was transduced by lentiviruses encoding HK1 D656A, sorted according to GFP expression, and the pool of GFP-positive cells was examined for HK1 expression by Western blotting and used for subsequent experiments.

### Cell proliferation assays

Cell viability in 96-well plates was measured by alamarBlue cell viability reagent (Thermo Fisher Scientific, Waltham, MA) following the manufacturer’s instructions. Cell proliferation in 96-well plates was also determined by the cell counting of the trypan blue-stained cells. All measurements were performed in three or more independent experiments, each performed in quadruplicate.

### NADPH/NADP and NAD/NADH ratio measurements

NADPH and NADP as well as NAD and NADH ratios were measured in 96-well plates by NADP/NADPH-Glo and NAD/NADH-Glo assays (Promega, Madison, WI), respectively. Briefly, 20,000 cells were seeded into each well in the respective growth media. After overnight attachment, the growth media were changed for the respective experimental media (DMEM supplemented with glutamine, with/without glucose and with/without metformin, 10% FBS) and cells were incubated for 6 hours at 37 °C in 5% CO_2_/95% air. Then the cells were processed following the manufacturer’s instructions and NADPH/NADP and NAD/NADH ratios were calculated and normalized to the cell counting performed after overnight attachment from additional wells using trypan blue. In this experiment, dialyzed FBS (Gibco A3382001, Thermo Fisher Scientific) was used for verification of outcomes obtained from experiments with standard FBS with 4 mM glucose.

The NADP/NADPH-Glo assay was based on the use of reductase. In the presence of NADP^+^ and NADPH, the reductase reduced a proluciferin substrate to luciferin, which was then quantified using the Ultra-Glo luciferase, and the luminescence was proportional to the amount of NADP^+^ and NADPH in the sample. To prepare a standard curve, we dissolved NADP^+^ with equal volumes of PBS, base solution with 1% DTB, 0.4N HCl, and 0.5M Tris base. The assay had a linear range of 10 nM to 400 nM of NADP^+^ and was specific for the phosphorylated forms. As a standard, we used NADP^+^ (MilliporeSigma, St. Louis, MO). All measurements were performed in three or more independent experiments, each performed in triplicate.

The NAD/NADH-Glo assay was based on the use of the NAD cycling enzyme, which was used to convert NAD^+^ to NADH. In the presence of NADH, reductase reduced a proluciferin reductase substrate to luciferin, which was quantified using Ultra-Glo recombinant luciferase and the luminescence signal was analyzed relative to the standard curve of NAD^+^ (MilliporeSigma), with control wells without NAD^+^ or cells used to determine the background luminescence. To prepare a standard curve, we dissolved NAD^+^ with equal volumes of PBS, base solution with 1% DTB, 0.4N HCl, and 0.5M Tris base. The assay had a linear range of 10 nM to 400 nM of NAD^+^ and was specific for the nonphosphorylated forms. All measurements were performed in three or more independent experiments, each performed in triplicate.

### Hexokinase activity assay in vitro

We induced HK1 expression by the addition of 1 mM IPTG and subsequently cultivated the cells for 16 h at 22 °C. Afterwards, we purified HK1 using HisTrap HP (GE Healthcare, Chicago, IL). We determined the HK1 activity using a coupled reaction with glucose-6-phosphate dehydrogenase to form NADPH with absorbance at 340 nm. The measurements were performed in the range of 0–2 mM glucose at 30 °C. All measurements were performed in three or more independent experiments, each performed in triplicate, and calculated *K*_M_ and *V*_max_ by nonlinear regression.

### Hexokinase activity assay in cellulo

Hexokinase activity was measured by a colorimetric hexokinase activity assay (Abcam, Cambridge, UK) following the manufacturer’s instructions. Briefly, 10^6^ of cells were seeded into each well in DMEM supplemented with 5.5 mM glucose and 2 mM glutamine, 10% FBS, and cells were incubated for 48 h at 37 °C in 5% CO_2_/95% air. Then, the cells were harvested, washed with ice-cold PBS, resuspended with ice-cold assay buffer, homogenized by pipetting, and centrifuged to remove insoluble materials, and supernatants were kept on ice until measured. The HK1 activity was measured using a coupled reaction with glucose-6-phosphate dehydrogenase to form NADH. NADH reduced a colorless probe to a product with strong absorbance at 450 nm. NADH was used as a standard; the lower assay limit was 0.1 mU/well. All measurements were performed in three or more independent experiments, each performed in triplicate.

### Antibodies and reagents

We used antibodies against HK1 (Cell Signaling, C35C4, 1:1000), HK2 (Cell Signaling, C65G5, 1:1000), β-actin (Santa Cruz, sc-47778, 1:200), c-Myc (Cell Signaling, D84C12, 1:1000), acetyl-CoA carboxylase (ACC) (Cell Signaling, C83D10, 1:1000), p-Ser79 ACC (Cell Signaling, D7D11, 1:1000), and lactate dehydrogenase A (LDHA) (Cell Signaling, C4B5, 1:1000). The plasmids pMD2.G (a gift from Didier Trono; Addgene plasmid #12259; http://n2t.net/addgene:12259; RRID:Addgene_12259) and psPAX2 (a gift from Didier Trono; Addgene plasmid #12260; http://n2t.net/addgene:12260; RRID:Addgene_12260) were used for production of lentiviral particles. Polyethylenimine 25K (Polysciences, Hirschberg an der Bergstrasse, Germany) was used for transfection. Lenti-X Concentrator (Takara, Kusatsu, Japan) was used for concentrating lentiviral stocks. To study chemosensitivity, metformin, rapamycin, and torin-1 (all from MilliporeSigma) were used.

### Mitochondrial membrane potential

Mitochondrial membrane potential (MMP) was measured by exposing cells to a JC-1 probe (Thermo Fisher Scientific). The medium was aspirated and cells were washed with PBS, trypsinized, and incubated in DMEM (Thermo Fisher Scientific; Gibco #A14430-01) with 5.5 mM glucose, 10% FBS for 2 hours at 37 °C in 5% CO_2_/95% air. Afterwards, the cells were incubated with PBS containing 1 μg/mL of JC-1 probe for 15 min at 37 °C in 5% CO_2_/95% air. The cells were washed with PBS and fluorescence was measured by the flow cytometer (BD FACSVerse, BD Biosciences). Unstained cells were used as the negative control; cells that were incubated with 100 μM trifluoromethoxy carbonylcyanide phenylhydrazone (FCCP) were used as a depolarization control. The results were visualized as the ratio of JC-1 aggregates (red fluorescence) towards the sum of red and green fluorescence in FlowJo (FlowJo, Ashland, OR).

### Mitochondrial mass

The medium was aspirated, cells were washed with PBS and trypsinized. Then, the cells were incubated in DMEM (Thermo Fisher Scientific; Gibco #A14430-01) with 5.5 mM glucose, 10% FBS for an hour at 37 °C in 5% CO_2_/95% air followed by a 10-min incubation in this medium containing 100 nM MitoTracker Green (Thermo Fisher Scientific). The cells were washed with PBS and fluorescence was assessed by flow cytometer (BD FACSVerse, BD Biosciences).

### ATP

ATP levels were measured by CellTiter-Glo luminescent cell viability assay (Promega, CA) following the manufacturer’s instructions.

### ROS and superoxide production

The medium was aspirated, cells were washed with PBS and trypsinized. Then, the cells were incubated in DMEM (Thermo Fisher Scientific; Gibco #A14430-01) with 5.5 mM glucose, 10% FBS for 5 hours at 37 °C in 5% CO_2_/95% air followed by a 15-min incubation in this medium containing 5 μM CM-H_2_DCFDA or 2 μM MitoSOX Red (Thermo Fisher Scientific) for total ROS or superoxide measurements, respectively. The cells were washed with PBS and fluorescence was assessed by BD FACSVerse (BD Biosciences). For the ROS measurements, the control TOV-112D cells that were incubated with 100 μM H_2_O_2_ were used as a positive control.

### RNA isolation, qRT-PCR, and immunoblotting

RNA was isolated using PicoPure RNA isolation kit. The isolated RNA was transcribed using SuperScript VILO cDNA synthesis kit and qRT-PCR was performed using the 7500 Fast Real-time PCR System. The housekeeping gene GAPDH was used to normalize in all the experiments. mRNA expression of the respective genes was assessed using gene-specific TaqMan probes (HK1: Hs00175976_m1; HK2: Hs00606086_m1; GAPDH: Hs99999905_m1) and TaqMan Fast Advanced Master Mix (all Thermo Fisher Scientific). All measurements were performed in triplicate.

For Western blotting, NuPAGE Bis-Tris 4 to 12% mini protein gels and NuPAGE MES SDS Running Buffer (both Thermo Fisher Scientific) were used. The western blots were quantified using ImageLab (Bio-Rad Laboratories, Hercules, CA).

### Extracellular acidification rate

The Glycolysis stress test was employed to measure glycolytic parameters on a Seahorse analyzer XFp (Agilent Technologies). The cells were plated at a density of 2 × 10^4^ cells per well in XFp tissue culture plates. The next day, the cells were washed and incubated for 30 min in XF Base medium, pH 7.4. Prior to the measurement, cells were counted using SpectraMax i3 (Molecular Devices, San Jose, CA). The following compounds were sequentially injected: 10 mM glucose, 2 μM oligomycin A, and 100 mM 2-deoxyglucose.

### Targeted metabolomics

The cells were processed as described [32]. The dried cell extracts were dissolved in 700 μL of cold MeOH:H_2_O (1:4) supplemented with 3 μL of norvaline (550 μg/mL). The mixture was evaporated in a glass vial at ambient temperature using a vacuum concentrator (Modul 4080C, Hanil Science Industrial, Incheon, Korea). The residue was dissolved in 30 μl of anhydrous pyridine and derivatized first with methoxyamine hydrochloride (25 mg/mL); incubation conditions: 2 h, 40 °C, shaking at 1500 rpm; followed by the second derivatization with N-tert-Butyldimethylsilyl-N-methyl-trifluoroacetamide with 50 μL of 1% tert-Butyldimethylchlorosilane; incubation conditions: 30 min, 70 °C, shaking at 1500 rpm. The reaction mixture was then diluted with a portion of 300 μL of hexane and analyzed for selected metabolites by two-dimensional comprehensive gas chromatography with mass detection (GC-MS) GC×GC-TOFMS (Pegasus 4D, Leco Corporation, St. Joseph, MI) controlled by ChromaTOF 4.5. A combination of non-polar Rxi-5SilMS (28.4 m × 0.25 mm, Restek, Bellefonte, PA), and polar BPX-50 (1.39 m × 0.1 mm, SGE) separation column was used for the GC×GC separation. Metabolites were detected as their oximated a tert-butylsilylated derivatives. Their identity was confirmed by using standards and by comparison of their mass spectra with those available in NIST Library and in-house build library. The obtained peak areas were normalized to the internal standard (norvaline) and the number of cells. Measurement of each clone was performed in five independent replicates.

### Xenograft experiments

To induce subcutaneous xenografts, 5 × 10^5^ of TOV-112D cells (HK1^−^, HK2^−^, controls with empty vector, and HK1 revertants) were suspended in 250 μL of their complete growth medium and implanted subcutaneously in the Crl:CD1-*Foxn1*^*nu*^ derivate mice under aseptic conditions. Cancer growth progression was assessed every 3 to 4 days by measurement of tumor diameters with a Vernier caliper; condition and body weight of the mice were also monitored. The mice were sacrificed when the longest tumor diameter reached 10 mm or at the end of the experiment at the post-injection day 30. In an experiment that focused on the effects of glucose-deficient diet, the mice were fed with Altromin C1073 (Altromin Spezialfutter, Lage, Germany), which is a complete glucose-deficient diet that provides 57% energy in a form of fat, 41% of energy in a form of proteins and 2% of energy in a form of saccharides. The experimental mice were provided with this diet 5 days prior to the xenotransplantation and were kept on the diet until the experiment termination. All reported experiments on animals were approved by the Ministry of Education, Youth and Sports of the Czech Republic under the protocol numbers MSMT-40542/2020-4 and MSMT-11189/2020-2.

### Statistical analysis

All measurements were performed in three or more independent experiments. Data are shown as means ± SE unless stated otherwise. Pearson’s correlation coefficient was used to estimate associations between continuous variables. For the analyses of the significance of Pearson’s correlations among multiple sets of blots, the Fisher’s *z* transformation of correlations was used. The Student *t*-test and one-way ANOVA with Student–Newman–Keuls post-test were used for statistical analyses. All the data were tested for the equality of variance and distribution normality; if the data did not fulfill these conditions, the Kruskal–Wallis ANOVA with Dunn’s post-test was used. The statistical analyses were conducted in SigmaPlot 12.0 (Systat Software, San Jose, CA); the Fisher’s *z* transformation was conducted in MAVIS 1.1.3 (http://kylehamilton.net/shiny/MAVIS/); the Fisher exact probability test in the 2 × 3 format was performed using the Freeman–Halton extension at http://vassarstats.net/fisher2x3.html. The GC×GC-MS data were analyzed by two-way ANOVA followed by Bonferroni *t*-test. Differences were considered statistically significant at *p* < 0.05.

## Results

### Expression of HKs in ovarian cancer

**Fig. 1:**
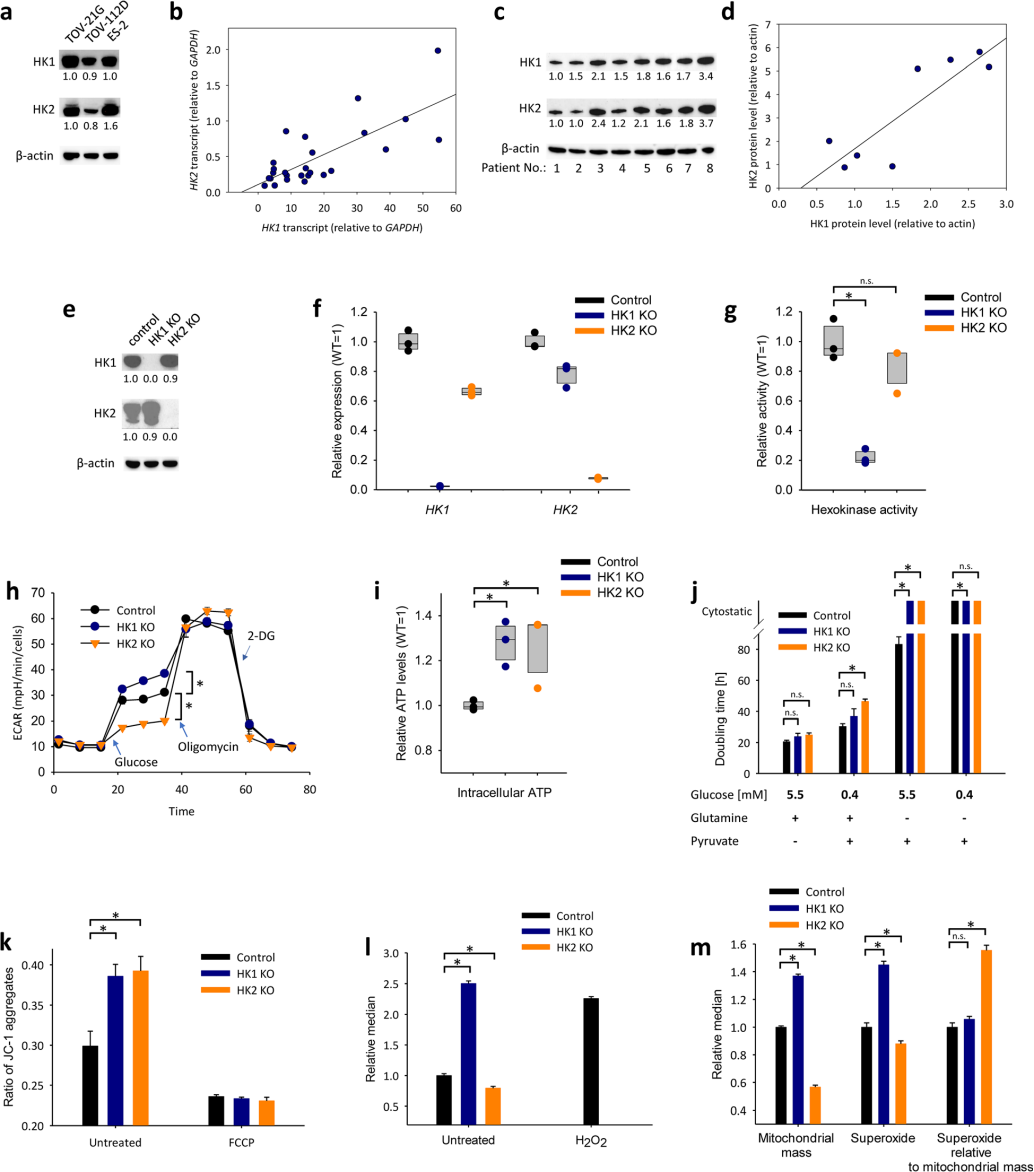
Expression of HK1 and HK2 in ovarian cancer and effects of HK1 and HK2 deletions. **a** Immunoblot of HK1 and HK2 in ovarian cancer cell lines. **b**, **c** The expression of HK1 and HK2 in primary ovarian cancer cells that were isolated and cultivated from high-grade serous ovarian carcinomas (HGSOC) of patients. The data are shown at the mRNA transcripts (**b**) and the protein (**c**). **b** mRNA: Pearson’s *r* = 0.747, *p* < 0.001, *n* = 26; protein: Fisher’s *z* transformed Pearson correlations of individual blots, fixed effects *r* = 0.938, *z* = 6.20, *p* < 0.001, random effects *r* = 0.936, z = 4.48, *p* < 0.001, *n* = 22. **c** Representative blot of hexokinases in primary HGSOC cancer cells. **d** The expression of HK1 and HK2 as analyzed by immunoblot of fibroblasts isolated from matched normal adjacent ovarian tissue of the examined ovarian cancer patients (Pearson’s *r* = 0.866, *p* = 0.005, *n* = 8). **e**, **f** Expression of HK1 and HK2 in HK1^−^ and HK2^−^ TOV-112D cells at the protein (**e**) and mRNA (**f**) levels. **g** The differences in hexokinase activity in the HK1^−^ or HK2^−^ TOV-112D cells. The differences in hexokinase activity were tested by one-way ANOVA (*F* = 6.0, df = 8, *p* < 0.001); Student–Newman–Keuls post-test *p* < 0.05 are indicated by asterisks. **h** Changes in extracellular acidification rates (ECAR) in the HK1^−^ or HK2^−^ TOV-112D cells. Asterisks indicate *p* < 0.05 in one-way ANOVA followed by Student–Newman–Keuls post-tests. **i** Changes in the intracellular ATP levels in the HK1^−^ or HK2^−^ TOV-112D cells. The differences in hexokinase activity were tested by one-way ANOVA (*F* = 33.1, df = 8, *p* = 0.04); Student–Newman–Keuls post-test *p* < 0.05 are indicated by asterisks. **j** Doubling time of the HK1^−^ or HK2^−^ TOV-112D cells in DMEM with altered glucose (5.5 mM or 0.4 mM), glutamine (2 mM or 0 mM), and pyruvate (1.2 mM or 0 mM) concentrations. Asterisks indicate *p* < 0.05 in one-way ANOVA followed by Student–Newman–Keuls post-tests. **k** Mitochondrial membrane potential visualized as the ratio of JC-1 aggregates (red fluorescence) towards the sum of red and green fluorescence in the HK1^−^ or HK2^−^ TOV-112D cells; cells that were incubated with 100 μM trifluoromethoxy carbonylcyanide phenylhydrazone (FCCP) were used as a depolarization control. Asterisks indicate *p* < 0.05 in one-way ANOVA followed by Student–Newman–Keuls post-tests. **l** Reactive oxygen species visualized by CM-H_2_DCFDA in the HK1^−^ or HK2^−^ TOV-112D cells. Data are shown relative to the control TOV-112D cells; the CM-H_2_DCFDA fluorescence following the incubation with 100 μM H_2_O_2_ is indicated as a positive control. Asterisks indicate *p* < 0.05 in one-way ANOVA followed by Student–Newman–Keuls post-tests. **m** Mitochodrial mass, superoxide, and superoxide relative to the mitochondrial mass in the HK1^−^ or HK2^−^ TOV-112D cells. Data are shown relative to the control TOV-112D cells (mitochondrial mass, superoxide). Asterisks indicate *p* < 0.05 in one-way ANOVA followed by Student–Newman–Keuls post-tests

To check for the expression of HK isoenzymes of ovarian cancer, we analyzed the expression at the mRNA and protein levels in ovarian cancer cell lines and primary cancer cells. Analysis of *HK1* and *HK2* mRNA expression in the Cancer Cell Line Encyclopedia [33] revealed that endometrial and ovarian cancer cell lines express both *HK1* and *HK2*. Moderate-to-strong expression of *HK1* and *HK2* (defined as > 10 TPM (transcripts per million)) was detectable in the vast majority of analyzed cell lines (Table S2). The majority of cell lines expressed more *HK1* transcripts than *HK2* (*HK1*:*HK2* ratio 2.3 ± 0.4), and the levels of *HK* transcripts correlated with one another (Fig. S1), which we further confirmed for selected three ovarian cancer cell lines at the protein level (Fig. 1a).

Correspondingly, in primary cancer cells cultivated from high-grade serous ovarian carcinomas, the levels of HK1 and HK2 correlated at the mRNA (Fig. 1b) and protein (Fig. 1c) levels. The protein levels of HK1 and HK2 correlated to a similar extent in the corresponding healthy tissues as well (Fig. 1d).

### Deletions of HK1 and HK2

Parallel expression of HK1 and HK2 in all analyzed cell lines and clinical patient samples indicates independent roles of these HKs. To identify the isoenzyme-specific roles, we prepared series of TOV-112D-derived clones with CRISPR/Cas9-induced deletion of HK1 or HK2 and confirmed this deletion at the protein level (Fig. 1e). Consistent with that, the HK1^−^ and HK2^−^ cells expressed only trace amounts of the target HK transcripts (Fig. 1f). At the protein level, the HK1 or HK2 deletion was not compensated by an increase in another HK isoenzyme. Although we noticed that a large part of HK1^−^ clones apparently increased the HK2 expression levels, we found that this HK2-mediated compensation of the HK1 deletion was correlated with the upregulation of c-Myc (Fig. S2). For further analyses of the effects HK1 and HK2 deletions, we used only clones, in which the protein expression of c-Myc was at a similar level as in the control clones. In the c-Myc-matched cell clones, the deletion of HK1 or HK2 was not compensated by the increase in activities of other HKs (Fig. 1g).

We next examined the effects of HK1 or HK2 deletions on the capacity of the glycolytic pathway after glucose starvation. The measurements of extracellular acidification rate (ECAR) revealed that the deletion of either HK1 or HK2 did not affect the glycolytic capacity. However, the glycolysis was paradoxically increased from in HK1^−^ (one-way ANOVA *F* = 146.6, df = 2, *p* < 0.001; Student–Newman–Keuls post-test *q* = 7.7, *p* = 0.002) and decreased in HK2^−^ cells (*q* = 16.0, *p* < 0.001) when compared to the control cells (Fig. 1h). Given that, the HK1 deletion led to a decrease of the glycolytic reserve, whereas the cells with HK2 deletion showed the increased glycolytic reserve when compared to the control cells (Fig. 1h). On the other hand, the HK1 and HK2 deletions were associated with increases in intracellular ATP (Fig. 1i).

Cancer cells exhibit high glycolytic activity during rapid proliferation even in the presence of normal oxygen concentrations in culture; therefore, we examined changes in doubling times following the HK1 and HK2 deletions. The doubling time was only marginally affected by HK1 and HK2 deletions when the cells were cultivated in the complete medium. However, the doubling times of HK1^−^ and HK2^−^ cells were prolonged in media with 0.4 mM glucose supplemented with glutamine and pyruvate. Importantly, in the medium without glutamine, the HK1 and HK2 deletions were cytostatic. Combined depletion of glutamine and glucose induced cytostasis in the control cells as well as the HK1^−^ and HK2^−^ cells (Fig. 1j).

As both, HK1 and HK2, are involved in mitochondrial homeostasis, we measured, whether the mitochondrial mass, mitochondrial membrane potential, and the ROS production are changed following their deletion. The HK1 and HK2 deletions were associated with the increase in mitochondrial membrane potential (Fig. 1k) by approximately one-third of its levels in the wild-type cells; the cells were completely depolarized when treated with 100 μM FCCP. The mitochondrial mass, total ROS levels, and superoxide increased only following the HK1 deletion but not following the HK2 deletion (Fig. 1l, m). However, as the superoxide probe is accumulating in the mitochondria, we normalized the superoxide levels to the mitochondrial mass. We found that there was no difference in superoxide relative to the mitochondrial mass between the control and HK1^−^ cells, but superoxide relative to the mitochondrial mass was higher in HK2^−^ cells compared to the control cells (Fig. 1m).

### Reversible HK1-specific effects on the response to metformin

**Fig. 2:**
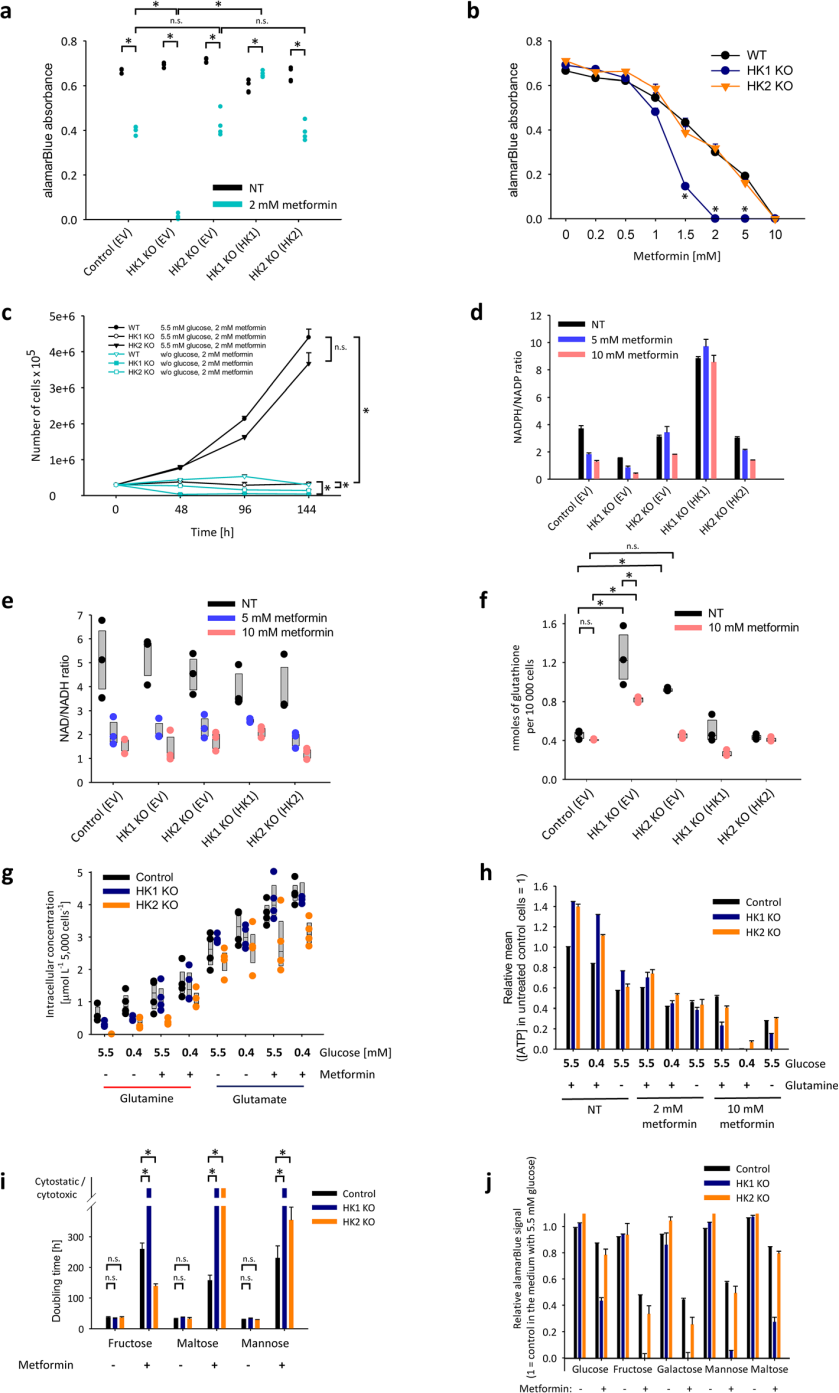
Reversible HK1-specific effects on the response of TOV-112D cells to metformin. **a** HK1 but not HK2 deletion induces the strong decline in the alamarBlue absorbance when cultivated with 2 mM metformin in the medium with only 0.4 mM glucose (one-way ANOVA *F* = 418.5, df = 9, *p* < 0.001; Student–Newman–Keuls post-test *p* < 0.001 are indicated by asterisks). **b** Concentration dependence of the decline in the alamarBlue absorbance in response to metformin treatment of the HK1^−^ or HK2^−^ TOV-112D cells that were cultivated in DMEM with 0.4 mM glucose for 36 h. The differences between the alamarBlue absorbance were tested by one-way ANOVA (*F* = 407.5, df = 23, *p* < 0.001); Student–Newman–Keuls post-test *p* < 0.001 are indicated by asterisks. **c** Cytostatic effects of the treatment with 2 mM metformin on HK1^−^ but not HK2^−^ or control cells in the presence of 5.5 mM glucose. **d–f** The NADPH/NADP (**d**), NAD/NADH (**e**), and glutathione (**f**) ratios in metformin-treated HK1^−^ or HK2^−^ TOV-112D cells that were cultivated in DMEM with 0.4 mM glucose for 36 h. The differences in glutathione levels were tested by one-way ANOVA (*F* = 23.7, df = 29, *p* < 0.001); Student–Newman–Keuls post-test *p* < 0.05 are indicated by asterisks. **g** The glutamine and glutamate ratios in the HK1^−^ or HK2^−^ TOV-112D cells that were cultivated in DMEM with 0.4 mM or 5.5 mM glucose in the presence or absence of 2 mM metformin for 24 h. **h** The ATP levels in metformin-treated HK1^−^ or HK2^−^ TOV-112D cells that were cultivated in DMEM with altered glucose (5.5 mM or 0.4 mM), glutamine (2 mM or 0 mM) in the presence or absence of 2 mM or 10 mM metformin for 24 h. **i** Doubling time of the HK1^−^ or HK2^−^ TOV-112D cells that were cultivated in DMEM with glucose replaced with 5.5 mM fructose, maltose, or mannose in the presence or absence of 2 mM metformin. The differences in doubling time were tested by one-way ANOVA (*F* = 46.6, df = 53, *p* < 0.001); Student–Newman–Keuls post-test *p* < 0.05 are indicated by asterisks. **j** AlamarBlue absorbance relative to that in the medium with 5.5 mM glucose generated by the HK1^−^ or HK2^−^ TOV-112D cells that were cultivated for 24h in DMEM with glucose replaced with 5.5 mM fructose, galactose, mannose, or maltose in the presence or absence of 2 mM metformin. Asterisks indicate *p* < 0.05 in one-way ANOVA followed by Student–Newman–Keuls post-tests

To inhibit the electron respiratory chain and therefore to inhibit the oxidation of glycolysis-derived pyruvate in mitochondria, we exposed the cells with HK1 or HK2 deletions to high-dose metformin. The HK1 deletion, but not the HK2 deletion, induced a dramatic decline in response when using alamarBlue assay after the 36-h-long treatment of cells with 2 mM metformin in the medium with only 0.4 mM glucose (i.e., FBS-derived glucose) when compared to identically treated control cells. The revertant of HK1 in the HK1^−^ cells restored the response to metformin to the level similar to the one observed in control cells (Fig. 2a). We next examined, whether the observed effect is concentration-dependent. Unlike the control and HK2^−^ cells, we found that HK1^−^ induces a more prominent signal decline when using alamarBlue assay even in lower metformin concentrations, with the 0.5 mM metformin in the medium with 0.4 mM glucose for 36 h (Fig. 2b). The metformin treatment was cytostatic, but not cytotoxic. During the 6-day-long incubation of cells in the presence of 2 mM metformin, the control and HK2^−^ cells grew to a similar extent when present in the medium without glutamine, while cytostasis was observed in HK1^−^ cells. When we repeated the same experiment with only 0.4 mM glucose, these conditions were cytostatic for control cells and cytotoxic for HK1^−^ and HK2^−^ cells (Fig. 2c).

### Redox imbalance in metformin-treated HK1^−^

Because the outcomes of the alamarBlue assay were not proportional to the number of living metformin-treated HK1^−^ cells, and because the alamarBlue assay outcomes depend on redox conditions in examined cells, we examined the cells for changes in redox balance. To address the likely causes of redox imbalance in metformin-treated HK1^−^ cells, we analyzed the NADPH/NADP ratios in metformin-treated cells (Fig. 2d). The NADPH/NADP ratio was more than twice lower already in untreated HK1^−^ cells. The metformin treatment caused a concentration-dependent decline of the NADPH/NADP ratio, which occurred proportionally in both the control and HK1^−^ cells. The HK2^−^ cells had the NADPH/NADP ratios in untreated cells similar to the control cells. In HK2^−^ cells, the metformin treatment did not induce any change in NADPH/NADP ratios unless the cells were treated with high metformin concentrations (Fig. 2d). Importantly, the HK1 revertant induced a strong increase in the NADPH/NADP ratios, and the differences compared to the wild-type were further potentiated when comparing the metformin-treated cells. In contrast, the HK2 revertant did not have any such effect (Fig. 2d).

In contrast to NADPH/NADP ratios, the NAD/NADH ratios in untreated and metformin-treated cells were only marginally affected by the HK1 or HK2 deletions (Fig. 2e). Note that both the NADPH/NADP and NAD/NADH ratios were analyzed in the presence of only 0.4 mM glucose. Glutathione levels responded to the HK1 or HK2 deletions, but did not explain the observed combined effect of HK1 deletion and metformin treatment (Fig. 2f). Also, the changes in the glutamine concentrations were similar in HK1^−^ and HK2^−^ cells; the glutamate levels were independent on the HK1 and HK2 deletions (Fig. 2g). Metformin treatment eliminated the increases in intracellular ATP in HK1^−^ and HK2^−^ cells. The HK1^−^ cells differed in ATP from the control and HK2^−^ cells only when treated with 10 mM metformin (Fig. 2h).

### Fructose as an alternative substrate of HKs

Glucose is not the only target of HKs and both HK1 and HK2 are capable to phosphorylate other hexoses [34]. When we replaced glucose with an equimolar amount of fructose, mannose, galactose, or maltose, the examined ovarian cancer cell lines survived and proliferated well when treated with metformin (Fig. 2i), which corresponds to the fact that the HKs are able to phosphorylate fructose [35]. Only the HK1 deletion (but not the HK2 deletion) sensitized them to the cytostatic and cytotoxic effects of metformin. The other monosaccharides did not rescue the effects of metformin following glucose withdrawal from the HK1^−^ cells (Fig. 2i, j). Only the maltose, a disaccharide composed of two glucose units, was able to partially rescue the alamarBlue signal (Fig. 2j) but did not prevent the cytostatic and cytotoxic effects of metformin following glucose withdrawal (Fig. 2i).

### Validation of HK1-induced effects using HK1 knock-down (KD)

**Fig. 3:**
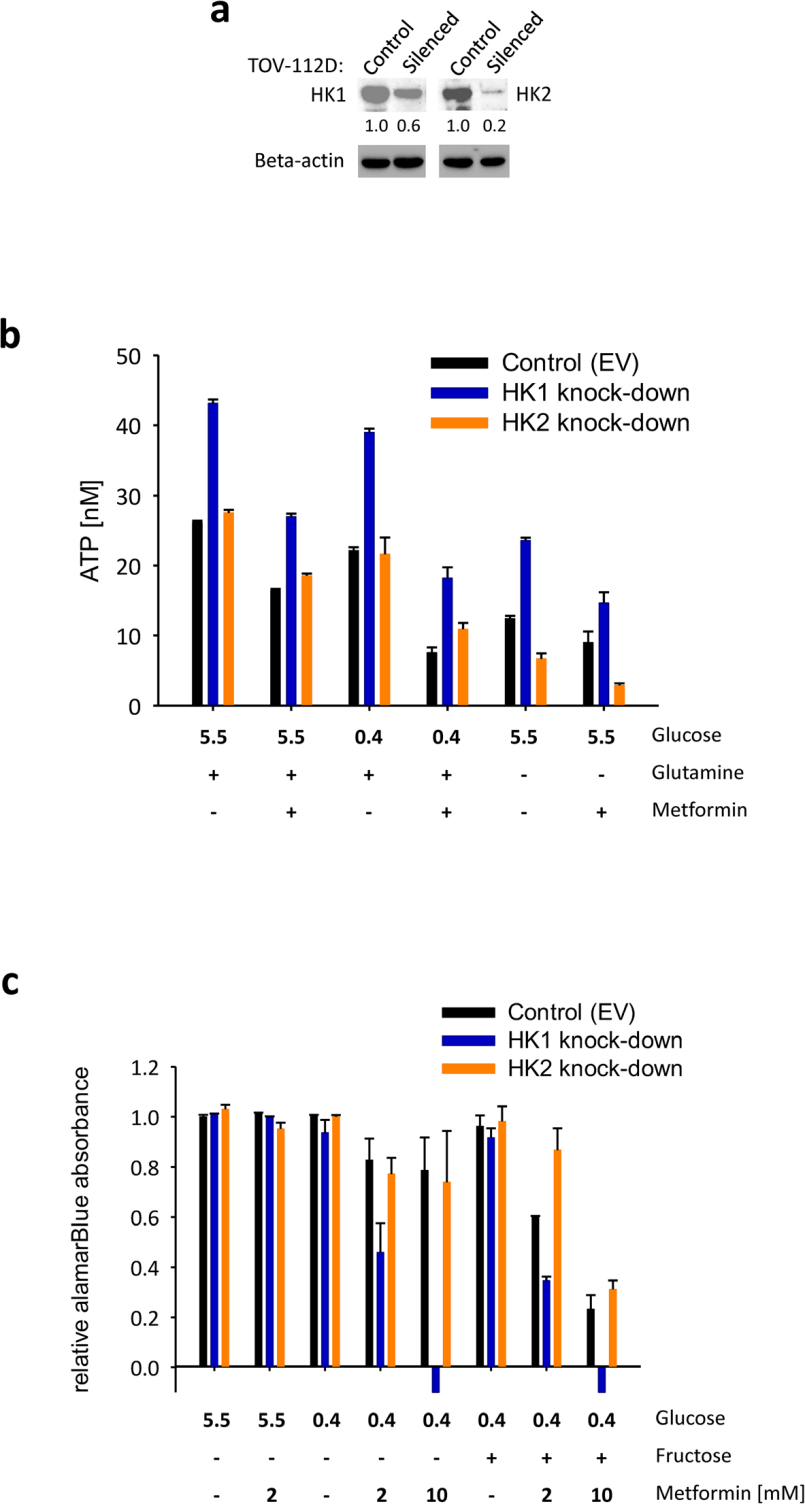
Validation of HK1-induced effects using HK1 knock-down (KD) in TOV-112D cells. **a** Immunoblot of HK1 and HK2 in HK1 or HK2 KD TOV-112D cells and in control cells with the empty vector (EV). **b** The ATP levels in metformin-treated TOV-112D cells with HK1 or HK2 KD that were cultivated in DMEM with altered glucose (5.5 mM or 0.4 mM), glutamine (2 mM or 0 mM) in the presence or absence of 2 mM metformin for 24 h. **c** AlamarBlue absorbance relative to that in the medium with 5.5 mM glucose generated by the HK1 or HK2 KD TOV-112D cells that were cultivated for 24 h in DMEM with 5.5 mM or 0.4 mM glucose or with 5.5 mM fructose in the presence or absence of 2 mM or 10 mM metformin

As the glycolysis-independent effects of HK1^−^ were surprising, we validated the key findings by preparing pools of cells with silenced expression of HK1 and HK2 (Fig. 3a). In HK1 KD, but not HK2 KD, we confirmed increased ATP levels under the basal conditions. These differences in ATP were retained following the glucose or glutamine withdrawal and when exposed to 2 mM metformin (Fig. 3b). Importantly, we confirmed the decline in the alamarBlue signal in HK1 KD, but not the HK2 KD, when the cells were grown in the medium with 0.4 mM glucose (Fig. 3c). The decline in HK1 KD was lower compared to that experienced in HK1^−^ when the cells were incubated with 2 mM metformin. However, the complete response was received when the cells were incubated with 10 mM metformin (Fig. 3c). Similar to the HK1^−^, the equimolar fructose did not rescue the effects of metformin following glucose withdrawal from the HK1 KD cells (Fig. 3c). The HK1 KD induced similar declines in the alamarBlue signal in ES-2 and TOV-21G cells when they were grown in the medium with 0.4 mM glucose with 10 mM metformin (Fig. S3). In the ES-2 and TOV-21G cells, fructose supplementation also did not rescue the effects of metformin (Fig. S3).

### Validation of HK1-induced effects in the mouse model

**Fig. 4:**
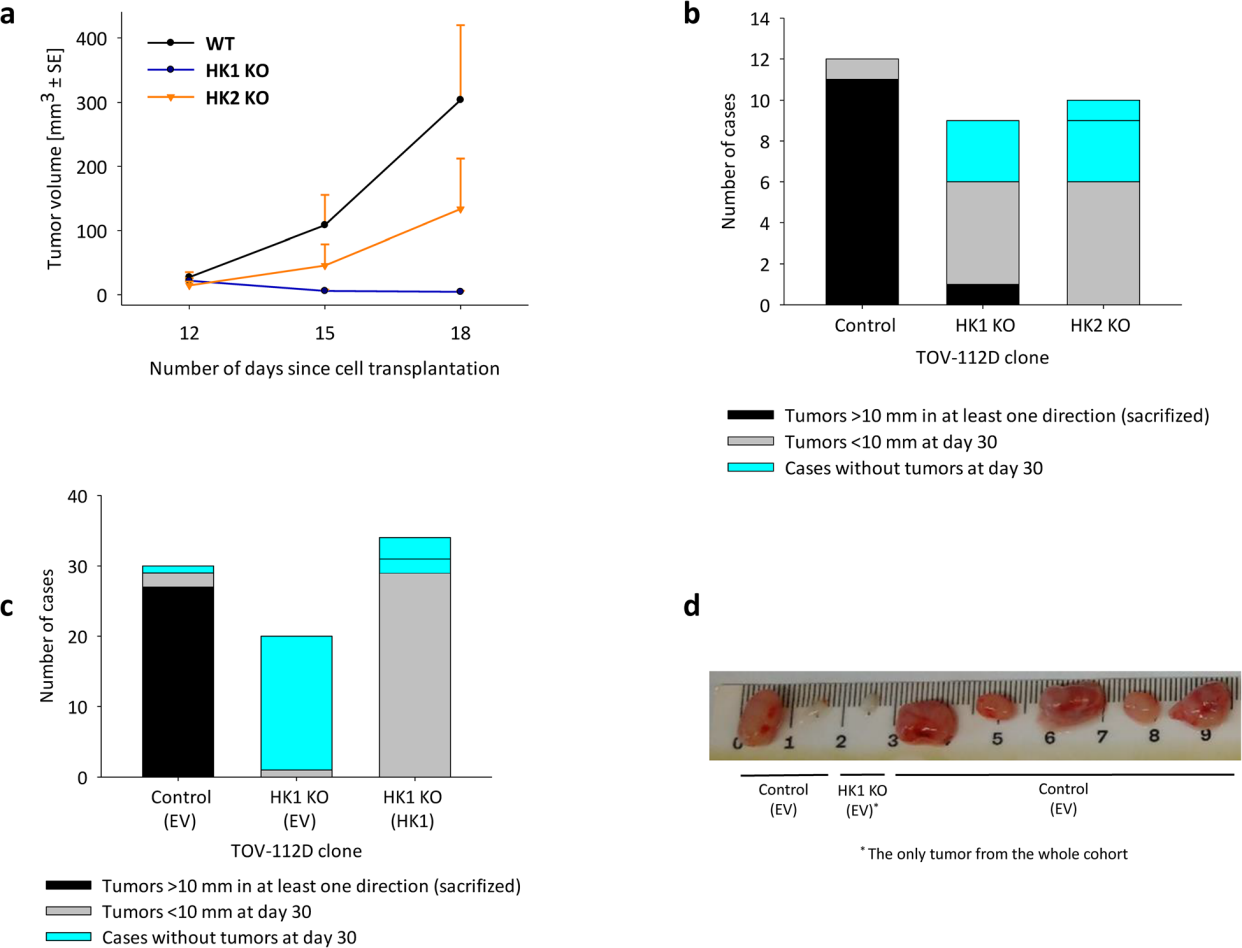
Validation of HK1-induced effects in the mouse model. **a** Tumor volume resulting from the subcutaneous xenotransplantation of TOV-112D control cells, HK1^−^ and HK2^−^ as evaluated at post-transplantation days 12, 15, and 18. **b** Endpoint outcomes of the experiment presented in Fig. 4a as evaluated at post-transplation day 30. **c** Endpoint outcomes of the subcutaneous xenotransplantation of the TOV-112D control cells, HK1^−^ cells, and HK1 revertants into mice that were fed with the glucose-deficient diet as evaluated at post-transplantation day 30. **d** Sample photographs of tumors in mice that were fed with the glucose-deficient diet after dissection, including the only tumor that developed in the cohort transplanted with HK1^−^ TOV-112D

To validate the observed effects in vivo, we performed two series of xenotransplantation experiments. Following the xenotransplantation of control, HK1^−^ and HK2^−^ TOV-112D cells, we evaluated the tumor volume at post-transplantation days 12, 15, and 18 (Fig. 4a). The tumor volumes were identical at day 12 but the xenotransplanted HK1^−^ cells did not grow any further and their volumes rather declined. In addition, the xenotransplanted HK2^−^ cells grew slower than the control cells, but both displayed exponential growth curves as opposed to the HK1^−^ cells (Fig. 4a). At the endpoint of the experiment (day 30), 11 out of the 12 control tumors grew to the maximum permitted size. In contrast, only one of the nine HK1^−^ tumors grew to the same size, five of the nine tumors (56%) grew to only a small size (< 10 mm in all directions) and in three mice, the HK1^−^ cells failed to engraft (Fig. 4b). The xenotransplanted HK2^−^ cells engrafted in all but one mouse but grew to the maximum permitted size in only six of the total 10 cases (Fig. 4b). The proportion of tumors that grew to the maximum permitted size differed significantly among the analyzed groups (Fisher exact test *p* < 0.001), and so was the proportion of tumors that failed to engraft (Fisher exact test *p* = 0.045).

The HK1^−^ cells were sensitive to glucose depletion in vitro despite the absence of the dominant hexokinase. Therefore, we xenotransplanted the TOV-112D control cells, HK1^−^ cells, and HK1 revertants into mice that were fed with the glucose-deficient diet, in which only 2% of the metabolized energy could be derived from any carbohydrates. Glucose deprivation highlighted the difference between the engraftment of control cells and the HK1^−^ cells, while the HK1 revertant completely abolished the engraftment inefficiency of the HK1^−^ cells (Fig. 4c). While only one of the 30 control cases failed to engraft and 90% of control cases grew to a maximum permitted size within the 30-day period, only one of the 20 HK1^−^ cases engrafted and formed only a small tumor at the endpoint of the experiment (Fig. 4d). In contrast, only three of the 34 HK1 revertant cases failed to engraft and 29 of the 34 HK1 revertant cases grew to a maximum permitted size before day 30 (Fig. 4c). The proportion of tumors that grew to the maximum permitted size differed significantly among the analyzed groups (Fisher’s exact test *p* < 0.001), and so was the proportion of tumors that failed to engraft (Fisher’s exact test *p* < 0.001).

### Catalytic-dead mutant of HK1

**Fig. 5:**
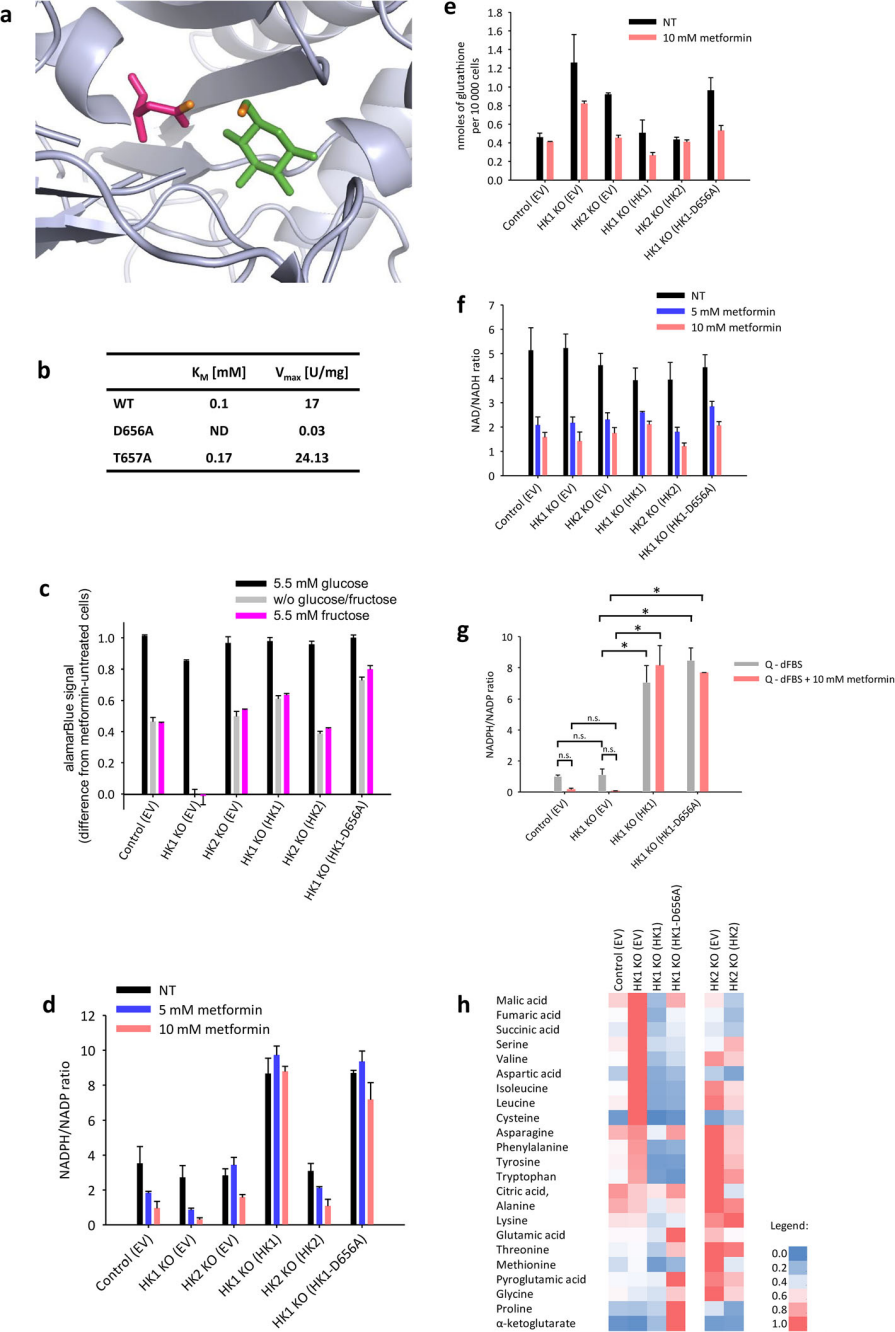
The catalytically dead variant of HK1. **a** Position of p.D656A and p.T657A variations in HK1, PDB structure 1HKB [36]. **b** *K*_M_ and *V*_max_ as measured in vitro for the WT HK1 and its p.D656A and p.T657A variations. **c** Relative alamarBlue absorbance generated by the HK1^−^ or HK2^−^ TOV-112D cells, HK1 and HK2 revertants and HK1 D656A knock-in in TOV-112D cells that were cultivated for 24 h in DMEM with 5.5 mM or 0.4 mM glucose or with 5.5 mM fructose in the presence or absence of 2 mM metformin. The data are displayed relative to alamarBlue absorbance of each respective cell clone in the respective condition without metformin. **d–f** The NADPH/NADP ratios (**d**), glutathione levels (**e**), and NAD/NADH ratios (**f**) in metformin-treated HK1^−^ or HK2^−^ TOV-112D cells, HK1 and HK2 revertants, and HK1 D656A knock-in in TOV-112D cells that were cultivated in DMEM with 0.4 mM glucose for 36 h. **g** The NADPH/NADP ratios HK1^−^ TOV-112D cells, HK1 revertants, and HK1 D656A knock-in in TOV-112D cells that were cultivated in DMEM without glucose and with dialyzed serum, with or without 2 mM metformin for 36 h. **h** Targeted GC×GC-MS analysis of changes in metabolites induced by changes in HK1 and HK2 in TOV-112D cells. Data are shown as relative values, where red indicates maximum concentration and blue indicates the lowest concentration of the respective metabolite in the six analyzed cell clones

We next aimed to verify whether the observed redox dysregulation of HK1^−^ is caused by catalytic or non-catalytic HK1 function. Therefore, we reverted the expression of the wild-type HK1, or, facultatively, the catalytically dead enzyme HK1 p.D656A (Fig. 5). To prepare catalytic dead mutants of HK1, we prepared the expression constructs of HK1 carrying either p.D656A or p.T657A variations (numbered according to the isoform 2, UniProt Acc. No. P19367-2) (Fig. 5a). For further experiments, we used the D656A mutant, which did not manifest a measurable *K*_M_ and which exhibited *V*_max_ lower by almost two orders of magnitude when compared to the wild-type. In contrast, the T657A mutant displayed higher *K*_M_ and *V*_max_ when compared to the wild-type (Fig. 5b).

The knock-in of HK1 D656A into HK1^−^ cells did not alter the total cellular HK1 activity when compared to the control HK1^−^ cells with empty vector (Fig. S4a) despite the HK1 protein levels in HK1 revertants and the HK1 D656A knock-in were higher than those in the control cells (Fig. S4b). The knock-in of HK1 D656A into HK1^−^ cells resulted in the complete reversal of the HK1^−^-induced alamarBlue signal decline under the presence of 0.4 mM glucose, both with and without 5.5 mM fructose (Fig. 5c). The NADPH/NADP ratios were similar in the wild-type HK1 revertants and HK1 D656A knock-in (Fig. 5d). Also, the glutathione levels were restored in the metformin-treated HK1 D656A knock-in; however, the glutathione levels were restored to only a partial extent in the untreated HK1 D656A knock-in cells (Fig. 5e). The HK1 D656A knock-in did not affect NAD/NADH ratios in untreated and metformin-treated cells relative to the HK1^−^ cells (Fig. 5f; Student–Newman–Keuls post-test *p* > 0.05 each for the control conditions, as well as the two metformin treatments).

### Targeted GC×GC-MS analysis

As the observed effects of HK1 deletion and loss of function extended beyond the expected glycolysis-related effects, we next analyzed the changes in amino acid levels using the GC×GC-MS approach. The GC×GC-MS analysis revealed that the HK1^−^ cells expressed high levels of l-aspartic acid, l-cysteine, and branched amino acids (BAAs) l-valine, l-leucine, and l-isoleucine. When we restored the HK1 expression, their levels sharply declined (Fig. 5h). The catalytically dead HK1 also restored the original levels of all these amino acids. The levels of TCA intermediates malate, succinate, and fumarate were also the highest in HK1^−^. The restored HK1 expression also induced declines in these amino acids; however, the trends towards their decline were not significant for malate or succinate when we introduced catalytically dead HK1. All the above reported changes were significant when tested by two-way ANOVA followed by Bonferroni *t*-test (*p* < 0.001 each; Fig. S5). Interestingly, these metabolites were affected much less by HK2 deletion; the HK2-induced changes of these metabolites were not considered statistically significant (*p* > 0.05 each).

### Confirmatory experiment using a glucose-free serum

To prove that the residual serum-derived glucose was dispensable for the observed effects, we measured the NADPH/NADP ratio in a medium that was supplemented with dialyzed glucose-free serum (Fig. 5g). We confirmed the significant increase in the NADPH/NADP ratio in the HK1 revertants in glucose-free conditions and regardless of metformin treatment. The knock-in of the catalytically dead HK1 had similar effects as the knock-in of the wild-type HK1 enzyme and ablated the differences in NADPH/NADP ratios induced by glucose withdrawal (Fig. 5g).

## Discussion

HK2 is considered a driver of tumorigenesis and metastasis [11–14], whereas HK1 is ubiquitously expressed in differentiated tissues [15]. Despite that, we revealed that HK2 and HK1 levels correlate in patient-derived ovarian epithelial cancer cells as well as fibroblasts from normal adjacent ovaries. Therefore, we assumed that HK1 plays an important role in ovarian cancer onset and progression. The HK1 or HK2 loss was previously suggested to cause the elevation of the other isoenzyme [37]. However, in agreement with previous chromatin immunoprecipitation experiments [38], we found that the expression of HKs is tightly correlated with c-Myc expression (Fig. S2). The expression of HKs is not compensated following the HK1 or HK2 loss as long as the c-Myc levels are kept constant.

The synthetic lethality in cancer cells has been previously reached by simultaneous inhibition of glycolysis, oxidative phosphorylation, and fatty acid oxidation [39]. Both HK1 and HK2 are inhibited by metformin, which may eventually lead to cell death [40]. The examined ovarian cancer cell lines survived and proliferated well when treated with metformin (Fig. 2c). Only the HK1 deletion (but not the HK2 deletion) sensitized them to the cytostatic and cytotoxic effects of metformin. The combined effects of HK1 deletion and metformin treatment were not tested previously and the analyses of HK1 expression levels were omitted in the majority of studies that examined the mechanisms of metformin action, e.g., [41]. In other types of cancer, HK2 is often the most abundant HK isoenzyme and may play a more important role, as higher chemosensitivity to metformin following HK2 depletion has been reported in lung and hepatocellular carcinomas [42, 43].

The effects of exposure of cell lines to metformin vary with a carbon source. Biguanides generally reduce mitochondrial ATP production [44, 45]. This reduction, together with cell growth suppression, is particularly prominent in the presence of glutamine and the absence of glucose. Observations of the effects of glucose withdrawal were performed using three cell lines, all of which express HK1 and HK2 [46, 47]. We found that the HK1 deletion alone is both necessary and sufficient to reduce ATP production in cells treated with high metformin concentrations in the presence of glutamine and the absence of glucose (Fig. 2h). This effect was absent when trace amounts of HK1 were present (Fig. 3b). In some cells, e.g., in MCF7 breast cancer cells but not the non-transformed C2C12 myoblast cells, limitation in glucose, glutamine, or pyruvate decreased ATP production rates 1.6-fold compared to cells with all these nutrients provided, mainly due to decrease in mitochondrial oxidative phosphorylation [48]. Insufficient ATP was suggested to be responsible for the growth-inhibitory effects of decreasing mitochondrial ribosomal proteins in respiratory conditions [49].

The TCA cycle substrates, like glutamine and glucose, are utilized differently in tumors and in vitro cultured cells [50–52]. Consistently, we observed little effects of the HK1 or HK2 deletion in vitro; however, the HK1 deletion substantially delayed the tumor growth in vivo (Fig. 4b). Moreover, when the mice were provisioned with glucose-free pellets, the xenotransplanted HK1^−^ cells but not the control cells completely failed to develop tumors (Fig. 4c). In vivo, these tumors were not exposed to a glucose free-environment but rather to constantly low glucose concentration. The near elimination of carbohydrates from the mouse diet causes that cycling involving alanine decreases, while glucose-lactate cycling persists and is based on lactate converted from glycerol [53]. Despite the gluconeogenesis, a low-carbohydrate diet slows the progression of certain cancers [54–58]. Therefore, HK1 is involved in yet undescribed nutrient-sensing and acquisition mechanisms with the capacity to shape the metabolic landscape of the tumor microenvironment.

The mechanisms of the glycolysis-independent role of HK1 are incompletely understood. Strikingly, the HK1 revertant but not HK2 revertant caused the increase of the NADPH/NADP ratio, even in the absence of glucose and presence of metformin (Fig. 5d). The cofactor NADPH, which is necessary for antioxidant defense and reductive biosynthesis, is primarily produced by the pentose-phosphate pathway (PPP), which branches off from glycolysis, and further by malic enzyme 1 (ME1) and isocitrate dehydrogenase 1 (IDH1). Since we confirmed our results in both low-glucose and glucose-free medium, we speculate that HK1 may induce a compensatory mechanism for the maintenance and increase of the NADPH/NADP ratio. However, ME1 and IDH1 may replace the PPP only partially [59]. The increase of the NADPH/NADP ratio may also be caused by fatty acid synthesis inhibition. The AMPK pathway may suppress fatty acid synthesis by the inhibition of acetyl-CoA carboxylases ACC1 and ACC2, thereby increasing NADPH generation by means of fatty acid oxidation [60]. We did not observe any changes in ACC phosphorylation; however, we observed a decline in ACC phosphorylation at Ser79 in metformin-treated HK1^−^ cells compared to control cells (Fig. S6). Furthermore, we did not observe any affection of NAD/NADH ratios (Fig. 5f).

Depletion of HK1 increased the cellular pool of both essential and non-essential amino acids. This could be caused by higher uptake of amino acids from medium, as well as limited proteosynthesis. Conversely, HK1 revertants decreased the pool of AAs. We hypothesized that this action of HK1 is unrelated to its catalytic activity; therefore, we confirmed this restoration of the pool of AAs by knock-in of catalytically dead HK1 mutant. Furthermore, note that the cells subjected to metabolomics analyses were cultivated in only 0.4 mM glucose. Given that, we observed that HKs are capable to rule proteosynthesis in the non-enzymatic manner.

In contrast, the presence of catalytically active HK1, but not HK2, was required for the maintenance of levels of TCA intermediates (malate, fumarate, and succinate), aspartate, and cysteine. All these metabolites were increased in HK1^−^ cells but remained unchanged in HK2^−^ cells. Aspartate is produced by transamination reaction between oxaloacetate and glutamate; thus its increase could be directly linked to the observed increases in malate and upstream TCA intermediates. Cysteine may be provided by increased uptake of cystine through the xCT/SLC7A11 antiporter, or even synthesized de novo. The effects on metabolism differed in HK1^−^ cells and in HK1 D656A knock-in cells. The catalytically dead HK1 was sufficient to restore the levels of TCA intermediates, aspartate, and cysteine. The catalytically dead HK1 also induced upregulation of α-ketoglutarate, glutamate, and its cyclization product pyroglutamate, but these three amino acids were at similar levels in control cells and in HK1^−^ cells. Whether increased cellular glutamate could be related to changes in the activity of xCT/SLC7A11 antiporter, or whether it reflects the need to feed the TCA cycle, remains unclear. Further research should also address the downstream effects of altered one-carbon flux on redox balance by providing feedback to yet unexplained mechanism of increase in NADPH/NADP ratio, which resulted from the HK1 overexpression in the non-enzymatic manner. Despite we provided here the conclusive evidence for the glycolysis-independent role of catalytically dead HK1, the mechanisms remain to be uncovered. Despite glycolysis being an essential and highly conserved metabolic pathway, all the glycolytic enzymes also have nonenzymatic moonlighting functions. These include the long-known structural functions of the lactate dehydrogenase B4 and α-enolase [61, 62], the RNA-enzyme-metabolite functions of the HKs, and other glycolytic enzymes [63, 64], yet unclear function of HKs in the cell nucleus [65, 66], immune receptor function of HK2 [67], apoptosis inhibition by HK2 [68, 69], and the glucose-sensing function of HKs [70]. The mechanism of the glucose-sensing function of HKs remains unclear and we speculate that the glucose-sensing function might be responsible for the observed differences between the cells with catalytically dead HK1 (“lack of glucose signal” present) and without any HK1 (“lack of glucose signal” absent).

As a limitation of the present study, it is worth to note that the examined cellular system (TOV-112D cell line and also the primary HGSOC cells) expressed predominantly HK1, while HK2 was expressed at a lower level. Therefore, the fact that HK2^−^ and HK2 KD cells displayed much milder phenotypes than the cells with altered HK1 expression could simply stem from the differences in expression levels of these two isoenzymes. Anyway, as we have shown that this difference in expression levels is characteristic for primary HGSOC cells, this cellular context is clinically highly relevant despite it was little explored in the past.

## Conclusions

The HK1 expression in ovarian cancer was both necessary and sufficient to sustain tumor progression, particularly when the cells were exposed to glucose starvation in parallel with high-dose metformin treatment. In ovarian cancer, HK1 alone is frequently a dominant hexokinase isoenzyme and appears to be sufficient to sustain tumor progression. Complete inhibition of HK1 is necessary to obtain the observed effects in combination with metformin, as they were recapitulated in HK1 KD cells only when higher metformin concentrations were used. Combined, the present study suggests the glycolysis-independent role of HK1 in tumor progression, which is involved in metformin resistance of the tumors.

## Supplementary Information

**Additional file 1.**

## Data Availability

All data are available in the main text or in the supplementary materials.

## Abbreviations

ACC: Acetyl-CoA carboxylase
AMPK: AMP-activated protein kinase
BAA: Branched amino acid
CDKN2A: Cyclin-dependent kinase inhibitor 2A
ECAR: Extracellular acidification rate
FCCP: Trifluoromethoxy carbonylcyanide phenylhydrazone
HGSOC: High-grade serous ovarian carcinoma
HK: Hexokinase
IDH1: Isocitrate dehydrogenase 1
KD: Knock-down
LDHA: Lactate dehydrogenase A
LKB1: Liver kinase B1
MDM2: Mouse double minute 2 homolog
ME1: Malic enzyme 1
MMP: Mitochondrial membrane potential
PPP: Pentose-phosphate pathway
TPM: Transcripts per million
