# Supplementary material for: Loss of hexokinase 1 sensitizes ovarian cancer to high-dose metformin
Source: Cancer Metab. 2021 Dec 11;9:41. doi: 10.1186/s40170-021-00277-2 (PMC8666047; doi:10.1186/s40170-021-00277-2)

SUPPLEMENTARY MATERIALS TO:

Loss of hexokinase 1 sensitizes ovarian cancer to metformin

Authors: Daniela Šimčíková[1], Dominik Gardáš[1], Kateřina Hložková[2,3], Martin Hruda[1,4], Petr Žáček[5], Lukáš Rob[1,4], Petr Heneberg[1]*

Affiliations:

[1]Charles University, Third Faculty of Medicine, Prague, Czech Republic

[2]CLIP – Childhood Leukaemia Investigation Prague, Prague, Czech Republic

[3]Department of Pediatric Hematology and Oncology, Second Faculty of Medicine, Charles University, Prague, Czech Republic

[4]University Hospital Kralovské Vinohrady, Prague, Czech Republic

[5]Charles University, Faculty of Science, BIOCEV, Vestec, Czech Republic

*Correspondence to: Petr Heneberg, Third Faculty of Medicine, Charles University, Ruská 87, CZ-100 00 Prague, Czech Republic, Tel: ++420 – 775 311 177, Fax: ++420 – 267 162 710, E-mail: petr.heneberg@lf3.cuni.cz.

Table S1. List of primers and sequences of sgRNAs.

***HK1* cDNA amplification and In-Fusion/XbaI restriction cloning into pHIV-EGFP**	
**Forward (5'→3')**	AGTTAACTATTCTAGAATGGACTGTGAGCACAGCC
**Reverse (5'→3')**	GCCCGGGTACTCTAGAGCTGCTTGCCTCTGTGCG
***HK2* cDNA amplification and In-Fusion/BamHI restriction cloning into pHIV-EGFP**	
**Forward (5'→3')**	ACCCGGGCTAGGATCCATGATTGCCTCGCATCTGC
**Reverse (5'→3')**	GGAGAGGGGCGGATCCTCGCTGTCCAGCCTCACG
***HK1* cDNA amplification and SacI/XhoI restriction cloning into pET-28a(+)**	
**Forward (5'→3')**	AAAGAGCTCATGGACTGTGAGCACAGCCTGAGTTT
**Reverse (5'→3')**	AAACTCGAGCTAGCTGCTTGCCTCTGTGCGTAACC
**Site-directed mutagenesis HK1 p.D656A (GAC→GCC)**	
**Forward (5'→3')**	GGTGGCTGTGGTCAACGCCACAGTGGGCACCATG
**Reverse (5'→3')**	CATGGTGCCCACTGTGGCGTTGACCACAGCCACC
**Site-directed mutagenesis HK1 p.T657A (ACA→GCA)**	
**Forward (5'→3')**	GGTGGCTGTGGTCAACGACGCAGTGGGCACCATG
**Reverse (5'→3')**	CATGGTGCCCACTGCGTCGTTGACCACAGCCACC
**sgRNA for HK1$^-$ preparation**	
**Sequence 22**	TTGCACCCGCAGAATTCGAA
**sgRNA for HK2$^-$ preparation**	
**Sequence 30**	CGTTGTGGCTCTGATCCGGA
**sgRNA for HK1 KD preparation**	
**Sequence 1**	GCCGCAACCAATGGGCGTGG
**Sequence 5**	CGCGCCGCAACCAATGGGCG
**sgRNA for HK2 KD preparation**	
**Sequence 1**	TGCGCACGTCACTGATCCGG
**Sequence 11**	AGCGATGATTGGCTGCGCCA

Table S2. Analysis of the *HK1* and *HK2* expression in the Cancer Cell Line Encyclopedia, experiment E-MTAB-2770 (31); n = 50 ovarian and endometrial carcinoma cell lines. The data are shown as the percentages of cell lines with the indicated expression level of the two transcripts.

**Transcript**	Low expression (≤10 TPM)	Moderate to strong expression (>10 TPM)
HK1	6% (HEC-1-A, HEC-108, COV644)	94%
HK2	2% (RMUG-S)	98%

Fig. S1. Expression of the *HK1* and *HK2* transcripts and their ratios in ovarian and endometrial cancer cell lines (n=50) in the Cancer Cell Line Encyclopedia, experiment E-MTAB-2770 (30). The levels of *HK* transcripts correlated with one another (Pearson correlation coefficient r = 0.345, p = 0.01). The data are plotted as the ratio of the *HK1* and *HK2* transcripts **(a)** and as the ratio of the *HK1* and *HK2* transcripts plotted against the expression levels of *HK1* **(b)**.

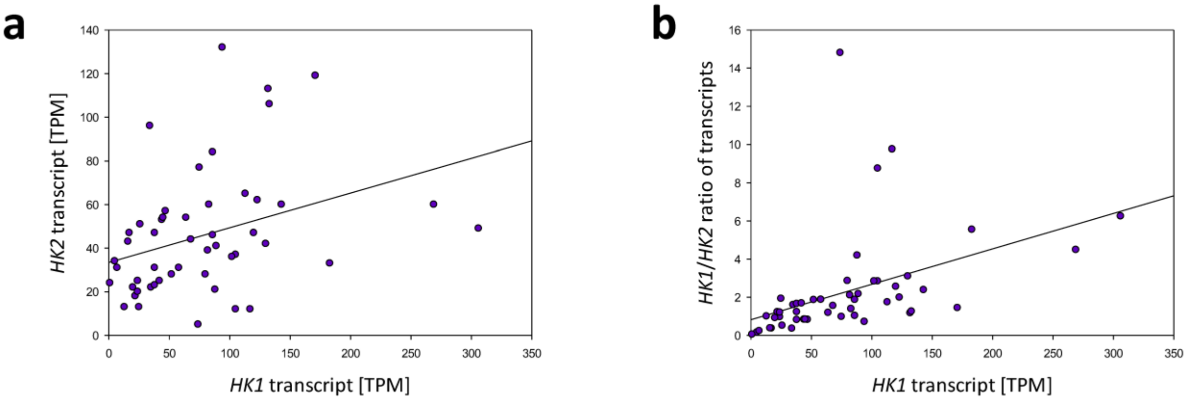

Fig. S2. Western blot of representative clones of TOV-112D cells that resulted from the CRISPR/Cas9-mediated attempt to knock-out the HK1. Note that the $HK1^{-}$ clones that had higher expression of HK2 had also upregulated c-Myc.

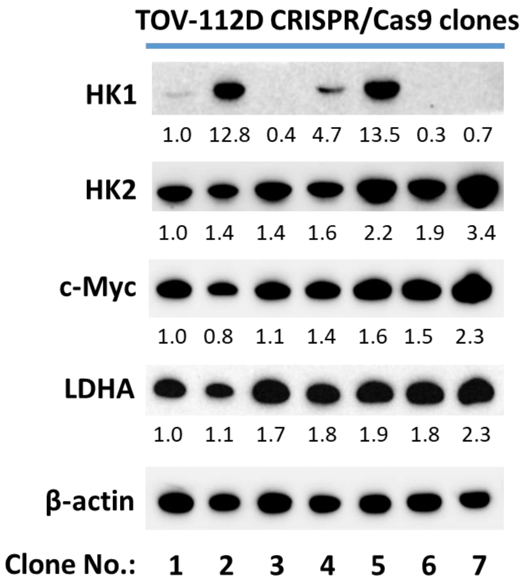

Fig. S3. AlamarBlue absorbance relative to that in the medium with 5.5 mM glucose generated by the HK1 or HK2 KD ES-2 **(a)** and TOV-21G **(b)** cells that were cultivated for 24h in DMEM with 5.5 mM or 0.4 mM glucose or with 5.5 mM fructose in the presence or absence of 2 mM or 10 mM metformin. EV = empty vector.

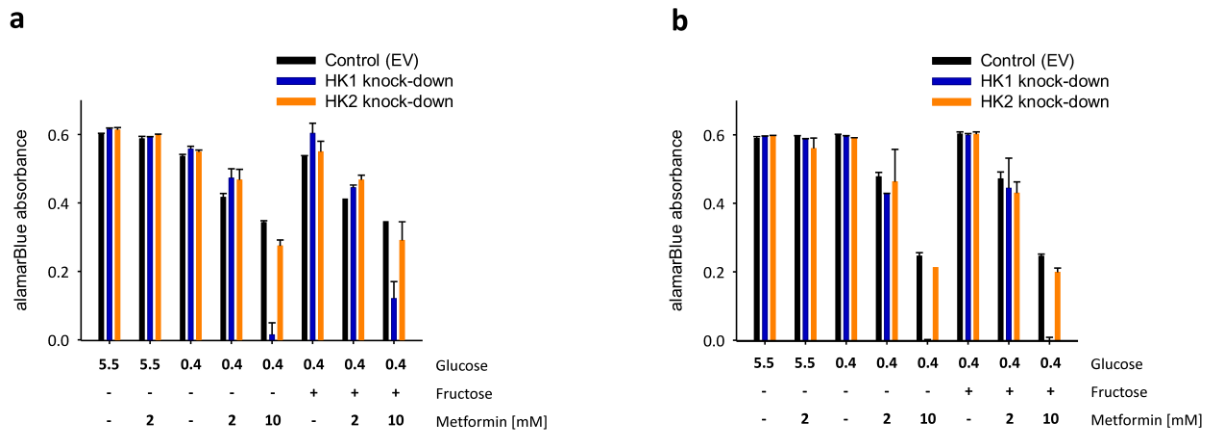

Fig. S4. Properties of TOV-112D clones with altered hexokinase (HK) expression or activity. (**a**) Relative total cellular hexokinase (HK) activity in the analyzed clones; the values are shown relative to the control (EV) cells. (**b**) Western blot of HK1 in the analyzed clones, including the HK1 revertant and HK1 D656A knock-in.

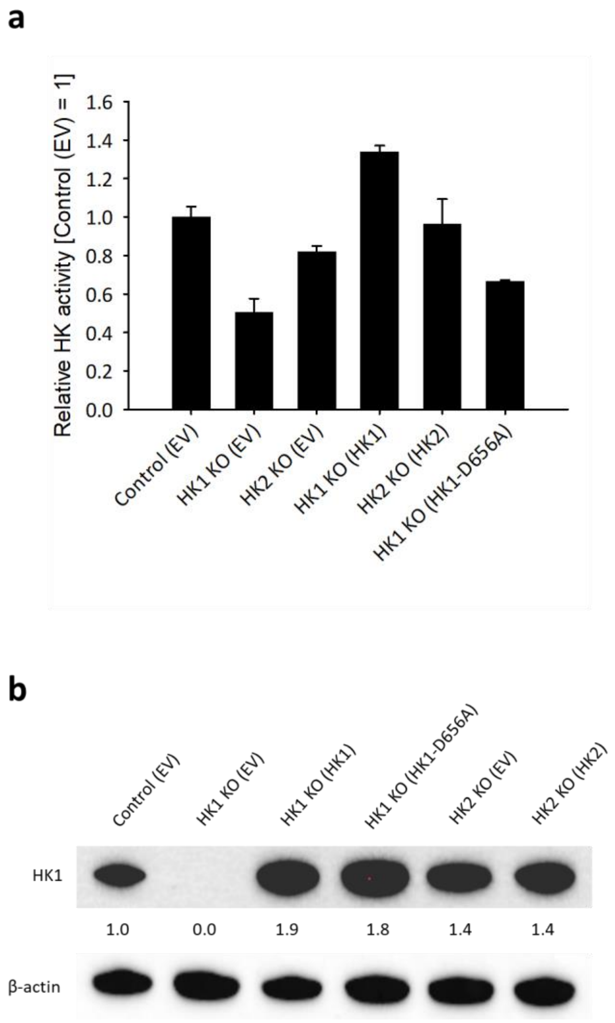

Fig. S5. Heatmap of outcomes of the two-way ANOVA analysis of changes in metabolites induced by changes in HK1 and HK2 using the GC-MS approach followed by Bonferroni *t*-tests. EV = empty vector. Dark red indicates the highly significant changes and blue indicates the changes of low significance for the respective metabolite among the six analyzed cell clones.

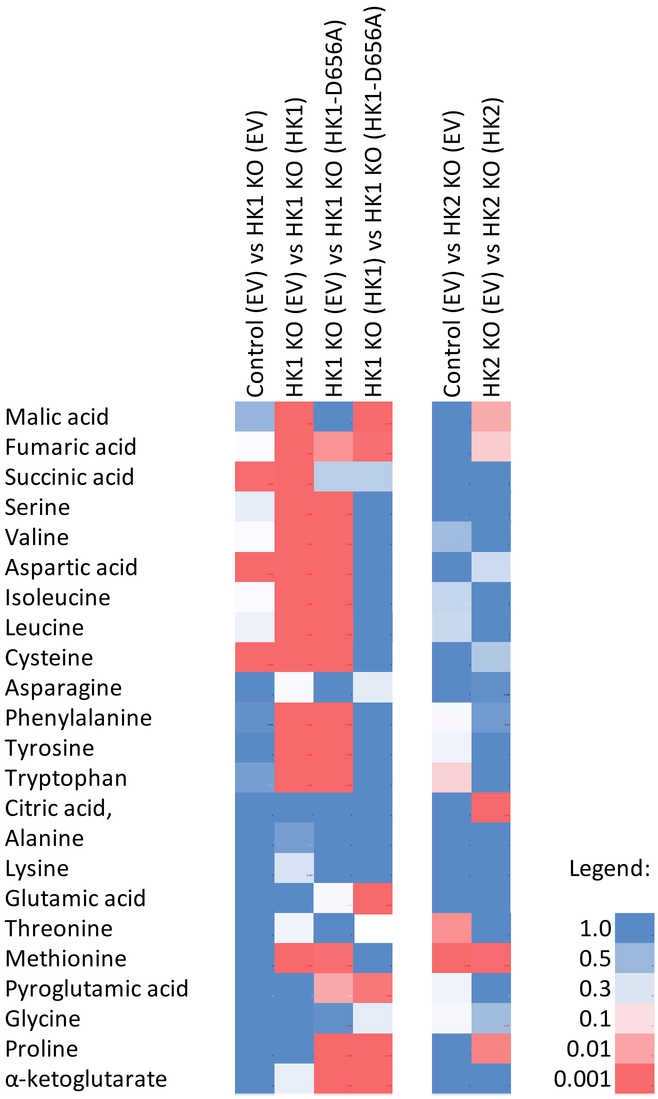

Fig. S6. Levels of ACC expression and ACC phosphorylation at Ser79 in TOV-112D HK1 KO cells and HK1 revertants cultivated in DMEM containing 5.5 mM glucose, 2 mM glutamine and 10% FBS, with or without 10 mM metformin for 6 h.

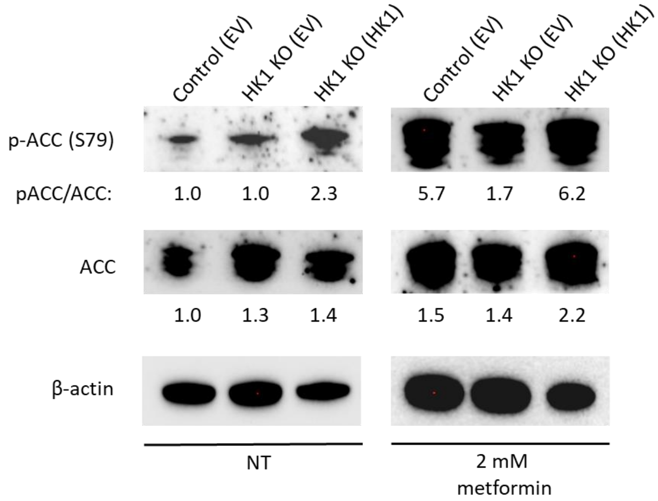

Supplement: Supplementary file 1 — Additional file 1. [file 40170_2021_277_MOESM1_ESM.pdf]
